# Association of lecithin-cholesterol acyltransferase activity and low-density lipoprotein heterogeneity with atherosclerotic cardiovascular disease risk: a longitudinal pilot study

**DOI:** 10.1186/s12872-018-0967-1

**Published:** 2018-12-05

**Authors:** Katsuaki Yokoyama, Shigemasa Tani, Rei Matsuo, Naoya Matsumoto

**Affiliations:** 1 Department of Cardiology, Nihon University Hospital, 1-6 Kanda-Surugadai, Chiyoda-ku, Tokyo, 101-8309 Japan; 2 Department of Health Planning Center and Cardiology, Nihon University Hospital, 1-6 Kanda-Surugadai, Chiyoda-ku, Tokyo, 101-8309 Japan

**Keywords:** LCAT, TRLs, LDL-particle size

## Abstract

**Background:**

Lecithin-cholesterol acyltransferase (LCAT) is believed to be involved in reverse cholesterol transport, which is known to play a key role in suppression of atherosclerosis. However, recent investigations have demonstrated that higher LCAT activity, measured in terms of the serum cholesterol esterification rate by an endogenous substrate method, is associated with increased formation of triglyceride (TG)-rich lipoproteins (TRLs), leading to a decrease in the low-density lipoprotein (LDL) particle size. The purpose of this hospital-based longitudinal study was to clarify the causal relationship between changes in the LCAT activity and changes in the LDL-particle size.

**Methods:**

The subjects were a total of 335 patients, derived from our previous study cohort, with one or more risk factors for atherosclerotic cardiovascular disease (ASCVD). For this study, we measured the LDL-particle size (relative LDL migration [LDL-Rm value]) by polyacrylamide gel electrophoresis in the subjects, along with the changes in the LCAT activity, at the end of a follow-up period of at least 1 year.

**Results:**

The results revealed that the absolute change (Δ) in the LDL-particle size increased significantly as the quartile of Δ LCAT activity increased (*p* = 0.01). A multi-logistic regression adjusted-analysis revealed that Δ LCAT activity in the fourth quartile as compared to that in the first quartile was independently predictive of an increased LDL-particle size (odds ratio [95% confidence interval]: 2.03 [1.02/4.04], *p* = 0.04). Moreover, the ∆ LCAT activity was also positively correlated with ∆ TRL-related markers (i.e., TG, remnant particle-like cholesterol [RLP-C], apolipoprotein B, apolipoprotein C-2, and apolipoprotein C-3).

**Conclusions:**

The results lend support to the hypothesis that increased LCAT activity may be associated with increased formation of TRLs, leading to a reduction in the LDL-particle size in patients at a high risk for ASCVD. To reduce the risk of ASCVD, it may be important to focus not only on the quantitative changes in the serum LDL-cholesterol levels, but also on the LCAT activity.

**Trial registration:**

UMIN (https://upload.umin.ac.jp/cgi-bin/ctr/ctr_reg_list.cgi) Study ID: UMIN000033228 retrospectively registered 2 July 2018.

## Background

Lecithin cholesterol acyltransferase (LCAT) is reported to be closely involved in reverse cholesterol transport (RCT), which is an anti-atherogenic process by which excess cholesterol is removed from the cells by high-density lipoprotein (HDL) and delivered to the liver for excretion [1].

However, according to the results of evaluation of the LCAT activity using the currently available assay methods, including both the exogenous and endogenous substrate methods, it appears quite likely that an increased LCAT activity is associated with the progression of atherosclerosis. Furthermore, several investigations have also suggested the existence of a positive correlation between increase in the serum levels of triglyceride (TG)-rich lipoproteins (TRLs) and elevation of the LCAT activity [2–4].

Recently, we reported that increased LCAT activity, as measured in terms of the serum cholesterol esterification rate by the endogenous substrate method, might be associated with a decrease in the LDL-particle size via its association with an increase in the serum levels of TRL-related markers in patients at a high risk for progression of atherosclerosis [5]. However, because this aforementioned study was designed as a cross-sectional study, and not as a longitudinal study or an interventional study, it was difficult to arrive at any definitive conclusions about the causal relationships based on the results.

Therefore, we designed this longitudinal study in an attempt to verify the hypothesis that elevation of the LCAT activity is associated with an increase in the serum levels of TRL-related markers, involved in disordered TG metabolism (i.e., TG, remnant particle-like cholesterol [RLP-C], apolipoprotein (apo) B, apo C-2, and apo C-3) [6], and is thereby involved in a reduction of the LDL-particle size.

The present study, with a longitudinal study design, as mentioned above, was undertaken to analyze the relationships between changes in the LCAT activity and changes in the LDL-particle size, particularly between elevation of the LCAT activity and diminution in the LDL-particle size.

## Methods

### Study design and populations

This study was designed as a hospital-based longitudinal study to investigate the relationship between the changes in the LCAT activity and changes in the LDL-particle size in the subjects of our previous cross-sectional study [5] who were available for additional measurements 1 year after completion of the previous study. The subjects underwent follow-up hematologic and blood biochemical tests at this institution at least 1 year after their participation in the completion of our previous study [5]. The primary endpoint was to evaluate the association between the absolute changes (∆) in the LCAT activity and the ∆ LDL-particle size using a multi-logistic regression analysis with adjustments for confounding factors, and the secondary endpoint was to investigate the associations between the ∆ LCAT activity and ∆ TRL-related markers, which are closely associated with TG metabolism.

The criterion for patient registration was the presence of one or more risk factors for atherosclerotic cardiovascular disease (ASCVD). The diagnostic criteria for the ASCVD risk factors were as follows: hypertension: systolic pressure ≥ 140 mmHg, diastolic pressure ≥ 90 mmHg, and/or current treatment with antihypertensive medication; diabetes mellitus (DM): fasting plasma glucose concentration ≥ 126 mg/dL, HbA1c ≥6.5%, and/or current treatment with anti-diabetic agents; dyslipidemia: serum LDL cholesterol (LDL-C) ≥140 mg/dL, serum TG ≥150 mg/dL, serum high-density lipoprotein cholesterol (HDL-C) ≤40 mg/dL, and/or current treatment with lipid-lowering medication; hyperuricemia: serum uric acid level ≥ 7.0 mg/dL and/or patient taking medications for control of blood uric acid levels; CKD: eGFR < 60 mL/min/1.73 m^2^, with the severity of chronic kidney disease (CKD) being determined based on the estimated glomerular filtration rate (eGFR) using the abbreviated Modification of Diet in Renal Disease (MDRD) Study equation, modified by a Japanese coefficient [7]; Obesity: body mass index (BMI) ≥25 kg/m^2^.

Patients were not enrolled if they met any of the following exclusion criteria: hepatic dysfunction (serum alanine aminotransferase and aspartate aminotransferase ≥2 times the upper limit of normal), known malignant disease, refusal to provide consent for participation in the study, diagnosis of acute coronary syndrome within 3 months prior to the study, and/or serum TG ≥400 mg/dL. The design and purpose of the study were approved by the Nihon University Surugadai Hospital Ethics Committee.

### Measurement of laboratory parameters

Fasting blood samples were collected in the early morning hours after the subjects had fasted overnight for 12 h. Serum LCAT activity was determined by a self-substrate method (SRL Co., Ltd., Tokyo, Japan), in which the serum free cholesterol is measured enzymatically after incubation of the serum with synthetic dipalmitoyl lecithin using a commercially available kit (Nescoat LCAT kit-S, Alfresa Pharma, Osaka, Japan) [8]. The LCAT activity measured by the present method showed good correlation with the values measured by the endogenous substrate method using gas-liquid chromatography [8], and the exogenous substrate method [9], and with the LCAT mass concentrations measured by enzyme-linked immunosorbent assay [10]. The serum total cholesterol (TC), HDL-C, and TG levels were measured using standard methods. Serum LDL-C levels were calculated using the Friedewald formula [11]. The serum RLP-C levels were measured using an immunoadsorption assay (SRL). The serum apo levels were determined using turbidimetric latex agglutination assays (Daiichi Pure Chemicals Co., Ltd., Tokyo, Japan). Serum high-sensitivity C-reactive protein (hs-CRP) levels were measured using a nephelometric assay (Behring Diagnostic Marburg, Germany).

### Measurement of the LDL-Rm value

**Fig. 1:**
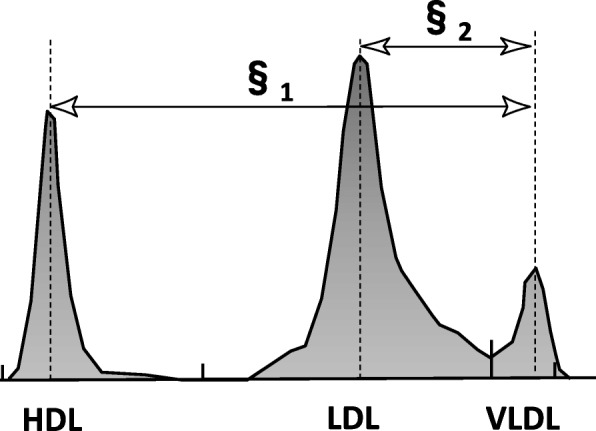
Measurement of LDL-Rm value by lipoprotein polyacrylamide gel disc electrophoresis. LDL-Rm = relative LDL migration, LDL-Rm value calculated from densitometer analysis of polyacrylamide disc gel electrophoresis; HDL = high-density lipoprotein; LDL = low-density lipoprotein; VLDL = very LDL LDL-Rm value are expressed as **§**_2_/**§**_1_

The relative LDL migration (LDL-Rm) value, an indicator of the LDL-particle size, was measured relative to the mobility value of LDL by polyacrylamide-gel electrophoresis (PAGE) using the LipoPhor system (Joko, Tokyo, Japan). The LDL-Rm value was calculated as the distance between the very LDL (VLDL) peak and the LDL peak divided by the distance between the VLDL peak and the HDL peak (Fig. 1). Several studies have reported that an LDL-Rm value of ≥0.40 suggests the presence of a large amount of small-dense (sd)-LDL in the LDL fraction [12]; a decrease of the LDL-Rm value indicates an increase in the LDL-particle size [13, 14].

In order to distinguish LDL-particle size phenotype A (large buoyant LDL) and phenotype B (sd-LDL), Hirano et al., established a cutoff value in LDL diameter of 25.5 nm as determined by PAGE, corresponding to an LDL-Rm value of 0.40. [13]. As described above, LDL-Rm value is often used for qualitative evaluation of the LDL-particle size in a clinical practice setting. Therefore, LDL-Rm value represents the average size of all LDL particles, which are aggregates of heterogeneously sized particles, and not the absolute amount of sd-LDL. However, the present study used LDL-Rm value as a continuous variable, which is keeping with previous drug interventional evaluating LDL-particle size conducted until now [15, 16].

### Statistical analysis

Data are expressed as the mean ± standard deviation for continuous variables and as percentages for discrete variables. For variables with a significantly skewed distribution, the data are expressed as interquartile ranges. In a subset analysis performed according to quartiles of the ∆ LCAT activity, we used analysis of variance (ANOVA) followed by Bonferroni’s adjustment for covariates if differences were detected. A multi-logistic regression analysis was performed to identify the variables associated with changes of the LDL-Rm value. Increase/decrease of the LDL-Rm value from the baseline was entered as the dependent variable, and the patient characteristics, risk factors for ASCVD, use/non-use of lipid-modifying drugs, and quartiles of Δ LCAT activity were entered as independent variables. Regression analysis was performed using linear regression, with estimation of the Spearman’s and Pearson’s correlation coefficients. All the statistical analyses were performed using the SPSS software program (SPSS Inc., Chicago, Illinois, USA) for Windows (version 12.0.1).

## Results

### Patients

The patient characteristics and laboratory profiles are shown in Tables 1 and 2. Among the subjects (*n* = 538) enrolled in our previous cross-sectional study [5], 335 subjects who were available for follow-up hematologic and blood biochemical tests at least 1 year after participation in completion of the previous study [5] were enrolled in the present study. Of these 335 patients, none had experienced any cardiovascular events during the 1-year interval from the previous study.

**Table 1:** Patients characteristics

	*n* = 335
Male / Female, *n* (%)	233 (70) / 102 (30)
Age (years)	62 ± 11
BMI (kg/m2)	24.8 ± 3.8
Hypertension, *n* (%)	224 (67)
Diabetes mellitus, *n* (%)	92 (28)
HbA1c (%)	6.1 ± 0.9
Current smoking, *n* (%)	26 (7.8)
Dyslipidemia, *n* (%)	269 (80)
eGFR (ml/min/1.73 m2)	69 ± 17
CKD Stage 3≥, *n* (%)	89 (27)
Number of risk factors	4.0 ± 1.5
Cardiovascular disease, *n* (%)	100 (30)
Coronary artery disease	87 (26)
Cerebral infarction	12 (3.6)
Aortic dissection/aortic aneurysm	4 (1.2)
Peripheral arterial disease	3 (0.9)
Concomitant drug, *n* (%)	
Anti-platelets	101 (30)
ACEs/ARBs	191 (57)
β blockers	77 (23)
Calcium channel blockers	183(55)
Lipid-modifying drugs	201(60)
Statins	179 (53)
Fibrates	8 (2.4)
Ezetimibe	15 (4.5)

*BMI* body mass index, *Hb* hemoglobin, *e-GFR* estimated glomerular flow rate, *CKD* chronic kidney disease, *ACEI* angiotensin converting enzyme inhibitor, *ARB* angiotensin receptor blocker

**Table 2:** Laboratory profile

	*n* = 335
Lipids	
TC (mg/dL)	188 ± 32
LDL-C(mg/dL)	100 ± 26
HDL-C (mg/dL)	57 ± 15
LDL-C/HDL-C ratio	1.89 ± 0.72
non-HDL-C (mg/dL)	131 ± 31
TRLs-related markers	
TG (mg/dL)	103 (76/157)
RLP-C (mg/dL)	4.1 (3.1/5.7)
apo B (mg/dL)	91 ± 21
apo C-II (mg/dL)	4.9 ± 1.9
apo C-III (mg/dL)	10.5 ± 3.3
LCAT activity (nmol/ml/hr/37 °C)	95 ± 20
LDL-Rm value	0.37 ± 0.03

*TC* total cholesterol, *LDL* low-density lipoprotein, *HDL* high-density lipoprotein, *TG* triglyceride, *RLP* remnant-like particle, *apo* apolipoprotein, *LCAT* Lecithin-cholesterol acyltransferase, *LDL-Rm* relative LDL migration

### Comparison of the Δ LDL-Rm value according to the Δ LCAT activity (as classified into quartiles)

**Fig. 2:**
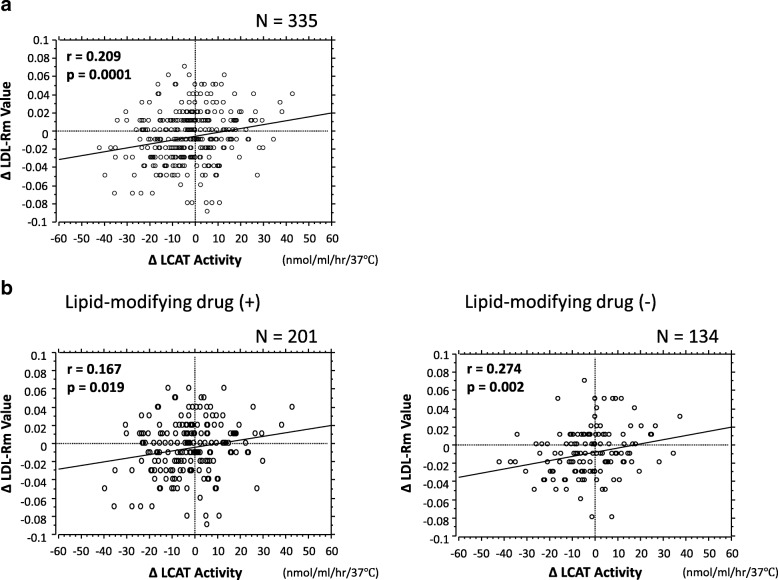
**a** Relationship between ∆ LCAT activity and ∆ LDL-Rm value LCAT = lecithin-cholesterol acyltransferase, LDL-Rm = relative LDL migration, Regression analysis was performed using linear regression and Spearman’s correlation coefficients. **b** LCAT = lecithin-cholesterol acyltransferase, LDL-Rm = relative LDL migration, Regression analysis was performed using linear regression and Spearman’s correlation coefficients

**Fig. 3:**
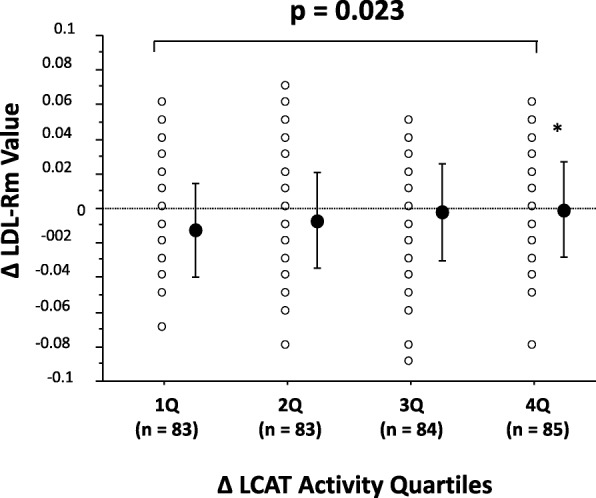
Comparison of ∆ LDL-Rm value according to ∆ LCAT activity (as classified into quartile) ∆ = absolute change from baseline, LDL-Rm = relative LDL migration, LCAT = lecithin-cholesterol acyltransferase. Error bar indicates mean ± standard deviation 1Q: − 41.8–− 10.0 nmol/ml/hr/37 °C, 2Q: − 9.0–− 1.7 nmol/ml/hr/37 °C, 3Q: 0–6.2 nmol/ml/hr/37 °C, 4Q: 6.3–43.4 nmol/ml/hr/37 °C. ANOVA and post hoc tests with Bonferroni correction were performed to test between-group differences. *p < 0.01 vs. 1Q

Among the subjects of this cross-sectional study [7], we investigated the association between the Δ LCAT activity, as classified into quartiles, and the Δ LDL-Rm values in the 335 patients who could be followed up for at least 1 year. The Δ LCAT activity was correlated positively with the Δ LDL-Rm value (Fig. 2a). Moreover, a positive correlation between Δ LCAT activity and Δ LDL-Rm value was also found in both patients receiving and not receiving lipid-modifying drug treatment (Fig. 2b). The Δ LCAT activity ranged from − 41.8 to 43.4 nmol/ml/hr/37 °C (mean ± SD: − 1.7 ± 13.8 nmol/ml/hr/37 °C, median; interquartile range in parentheses: − 1.5 (− 9.9/6.3 nmol/ml/hr/37 °C). The patients were divided into quartiles according to the Δ LCAT activity, as follows: first quartile, − 41.8 to − 10.0 nmol/ml/hr/37 °C (*n* = 83), second quartile, − 9.9 to − 1.7 nmol/ml/hr/37 °C (*n* = 83), third quartile, − 1.6 to 6.2 nmol/ml/hr/37 °C (*n* = 84), and fourth quartile, 6.3 to 43.4 nmol/ml/hr/37 °C (*n* = 85). The Δ LDL-Rm value increased with increasing quartile of Δ LCAT activity, the differences (*p* = 0.023) (Fig. 3) indicating that the higher the Δ LCAT activity, the smaller the Δ LDL-particle size.

### Multi-logistic regression analysis to identify variables that were independently correlated with the changes of the LDL-Rm value, an estimate of the LDL-particle size

To investigate the relationships between elevation of the LCAT activity and diminution in the LDL-particle size, multiple logistic regression analysis in the 335 patients conducted with adjustments for age, gender, risk factors for ASCVD, and history of use of lipid-modifying drugs revealed that Δ LCAT activity in the fourth quartile was an independent predictor of increased LDL-RM values, namely, smaller LDL-particle sizes (Table 3). However, no association between increased Δ LCAT activity and decreased Δ LDL-Rm value was found in the cases with serum LDL-C < 100 mg/dL, (1Q vs. 4Q, OR: 1.711; 95% CI: 0.649–4.510; *p* = 0.277).

**Table 3:** Multi-logistic regression analysis to identify variables that were independently correlated with the changes of the LDL-Rm value, an estimate of the LDL-particle size

Variable	OR	95% CI		*n* = 335
		Upper	Lower	*p* value
Age	0.973	0.951	0.995	0.018
Male gender	1.108	0.645	1.903	0.710
BMI	0.991	0.929	1.056	0.776
Current smoking	0.625	0.240	1.627	0.336
Diabetes mellitus	0.822	0.475	1.422	0.483
Hypertension	0.618	0.367	1.041	0.484
Dyslipidemia	1.704	0.768	3.781	0.190
Lipid-modifying drugs	1.368	0.734	2.552	0.324
∆LCAT 1Q	Reference			
∆LCAT 2Q	1.498	0.738	3.039	0.263
∆LCAT 3Q	1.784	0.884	3.601	0.163
∆LCAT 4Q	2.028	1.019	4.035	0.044

*OR* odds ratio, *CI* confidence interval, *BMI* body mass index, *∆* absolute change from baseline, *LCAT* lecithin-cholesterol acyltransferase, *Q* quartile

### Relationship between Δ LCAT activity and Δ TRL-related markers

**Fig. 4:**
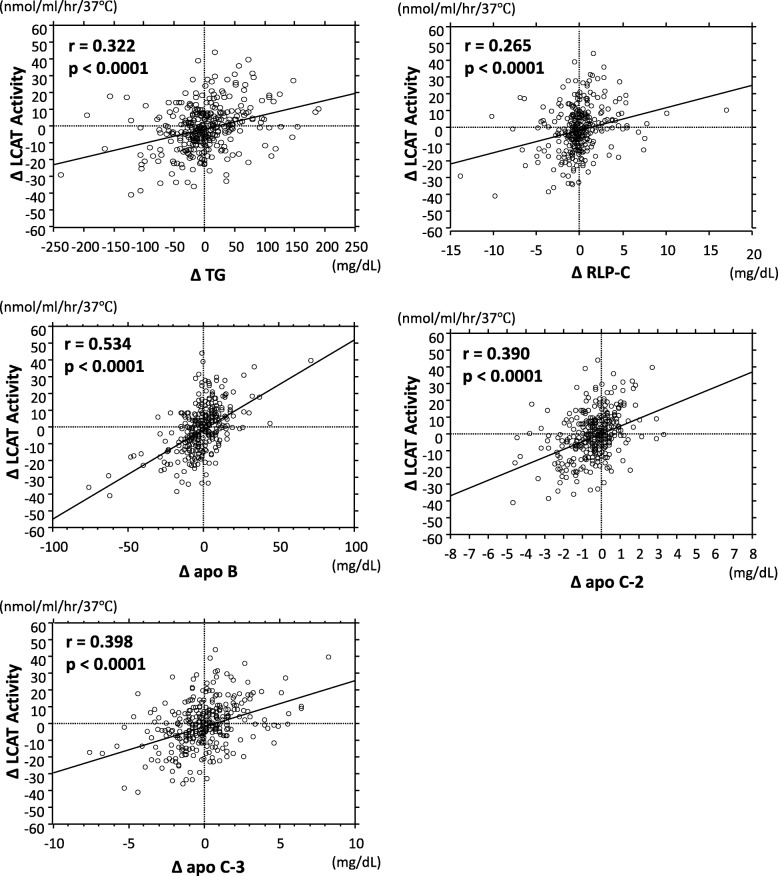
Relationship between ∆ LCAT activity and ∆ TRL-related markers ∆ = absolute change from baseline, LCAT = lecithin-cholesterol acyltransferase, TRL = triglyceride–rich lipoprotein. Regression analysis was performed using linear regression and Pearson’s correlation coefficients

Investigation of whether changes in the serum levels of TRL-related markers specifying the LDL-particle size might be correlated with changes in the LCAT activity was then pursued, inasmuch as the research hypothesis that elevation of LCAT activity was an independent predictor of diminution of the LDL-particle size had been verified. Figure 4 shows the simple correlations between the Δ LCAT activity and Δ serum TRLs-related markers. Positive correlations were noted between the Δ LCAT activity and all Δ TRL-related markers.

## Discussion

This longitudinal study yielded the following findings. Elevation of LCAT activity, as measured in terms of the serum cholesterol esterification rate by the endogenous substrate method, was associated with a decrease in LDL-particle size, which exhibit potent atherogenic activity, and increased LCAT activity may be depend on increased serum levels of TRL-related markers in patients at high risk for ASCVD.

The present longitudinal study on the correlations among LCAT activity, serum levels of TRL-related markers and the LDL-particle size was conducted in the same subject cohort as that in our previously reported cross-sectional study [5], and demonstrated that an increase in the LCAT activity may bring about elevations of the serum TRL-related markers, i.e., indicators of disordered triglyceride metabolism, and down-sizing of the LDL-particle size, and thereby possibly trigger an atherosclerogenic effect rather than exerting an anti-atherosclerogenic effect associated with activation of the RCT system. This is consistent with the findings reported heretofore [2–4].

Importantly, only cross-sectional study was unable to establish a causal relationship between the results, but the results of the two studies with different (cross-sectional [5] and longitudinal) designs taken together strongly suggests that an increase of the LCAT activity was associated with a decrease of the LDL-particle size.

It has been reported that the increased serum cholesterol esterification generated by the LCAT reaction may be the result of an increase in TRLs, and that it may, therefore, represent potentially decreased LDL-particle size through activities of cholesteryl ester transfer protein and hepatic lipase [3, 17, 18]. These reports provide support for what have been demonstrated in the present study, i.e., that elevation of LCAT activity constitutes a determinant of diminution of the LDL-particle size and that a positive correlation exists between absolute changes in LCAT activity and changes in the TRL-related markers from the baseline.

However, the RCT system represents a complicated network mediated not merely by the bioactivity of LCAT, but by the activities of many other enzymes as well, to exert an anti-atherosclerogenic effect of HDL in toto [19, 20]. We may have to interpret the present findings, solely focused upon the LCAT activity, TRL-related markers and LDL-particle size, as implying nothing more than a demonstration of a causal relationship of these three factors with atherosclerogenic effect.

It has also been reported that the anti-atherosclerogenic effect of RCT is dependent on the state of lipid metabolism which has a profound bearing on progression of atherosclerosis. Several investigations suggest that in the presence of abnormal lipid metabolism that can cause ASCVD, RCT may be activated and LCAT may stimulate esterification of free cholesterol, possibly resulting in changes in the direction toward suppressed progression of atherosclerosis [20–22]. In support of these reported findings, a negative correlation between the plaque volume assessed by intravascular ultrasonography and the LCAT mass concentration has been documented in coronary artery disease patients in recent years, and the authors have suggested that LACT activity is up-regulated with a consequent facilitation of RCT, leading to a reduction in coronary artery plaques in patients with coronary artery disease [23].

There are few reports yet of large-scale prospective cohort studies investigating LCAT activity in relation to the prognosis of coronary artery disease. Most recently, in a prospective cohort study carried out in the general population in Japan, a positive correlation was observed between the LCAT activity, measured in terms of the serum cholesterol esterification rate assessed by the endogenous substrate method, and the serum levels of the TRLs, and the group with elevated LCAT activity showed a significantly higher incidence of sudden death and coronary artery disease [24]. This may provide evidence in support of our study results, although the cited study did not examine the relation between TRLs and the LDL-particle size.

Interestingly, what would deserve special mention here is that the coefficient of correlation between the Δ LCAT activity and Δ LDL-Rm was higher in the patient group receiving lipid-modifying drugs (mostly statins) as compared to that in the group that was not receiving lipid-modifying drugs (Fig. 2-2). Lipid-modifying drugs seem likely to suppress the downsizing of the LDL-particle size associated with elevation of the LCAT activity, although this may only be stated parenthetically in the presence of intergroup differences in sample size and patient background characteristics.

**Fig. 5:**
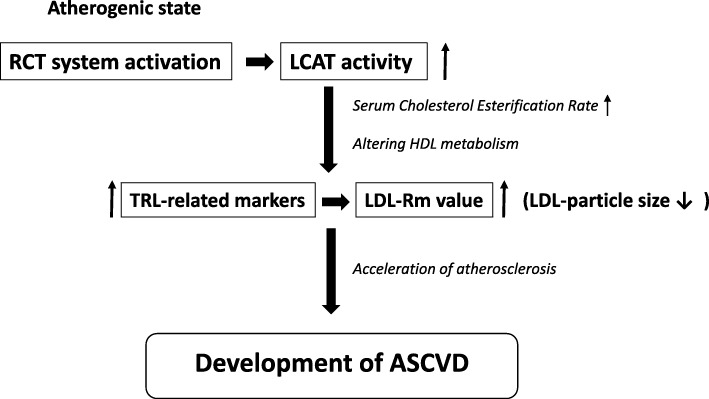
A possible association of LCAT activity and LDL-Rm value, an indicator of LDL-particle size, with the development of ASCVD in this study. In an atherogenic state, RCT system might be activated. Accordingly, increased LCAT activity measured as a serum cholesterol esterification rate by the endogenous substrate method might be associated with altering HDL metabolism, resulting in an increase in the serum levels of TRL-related markers and a decrease in LDL-particle size (i.e. an increase in LDL-Rm value), leading to the development of ASCVD. However, further investigations are necessary for the elucidation of the precise mechanism involved in atherosclerosis associated with increased LCAT activity and decreased LDL particle size. LCAT = lecithin-cholesterol acyltransferase, LDL = low-density lipoprotein, LDL-Rm = relative LDL migration, ASCVD = atherosclerotic cardiovascular disease, RCT = reverse cholesterol transport, HDL = high-density lipoprotein, TRL = triglyceride–rich lipoprotein

In the preceding investigation conducted as a cross-sectional study, a correlation was shown to exist between increase of LCAT activity and diminution of the LDL-particle size in patients with serum LDL-C levels of < 100 mg/dL. In this study, however, which was conducted using the data of patients at high risk for ASCVD, a multivariate logistic regression analysis indicated that in patients with serum LDL-C levels of < 100 mg/dL, who are assumed to have only a low tendency towards progression of atherosclerosis, increase of the LCAT activity had no impact on the LDL-particle size. This would be interpreted as being due to a decline in the statistical detection rate owing to the small case-sample size of this study. We propose to further verify the present research hypothesis through increasing the case-sample size. Figure 5 illustrated our hypothesis that increased LCAT activity might be associated with an increase in TRL-related markers and a reduction of LDL-particle size, possibly leading to the development of ASCVD.

### Study limitations and clinical implications

First, this study did not incorporate analysis of changes in the properties of the atherosclerotic lesions by diagnostic imaging [25]. Secondly, interventional studies using drugs which have the potential to modify LCAT activity (e.g., lipid-modifying drugs) may be useful for clarifying the relationship between LCAT activity and lipid metabolism [26]. It is expected that interventional studies with various drugs will be conducted in the future to examine the effect of LCAT on the progression of arteriosclerosis. We previously reported, in our original article, a patient with obesity who responded to prescribed dietary control with weight reduction accompanied by lowering of the serum levels of TRLs and LCAT activity, and a decrease of the LDL-Rm value [27]. Similarly, there have been reports of lowering of the serum levels of TRLs and sd-LDL and LCAT activity associated with controlled weight reduction [28]. Thus, further evidence needs to be accumulated to establish the exact relation between the serum LCAT activity and LDL-heterogeneity. Thirdly, this study does not demonstrate the relationship between LCAT activity and clinical indices and/or outcomes, because it was only a pilot study. Finally, we have pursued the argument in this paper on the premise that LCAT activity represents an atherosclerosis-promoting factor; however, we may have to discuss with great deliberation whether LCAT facilitates, or in fact, suppresses atherosclerosis on the ground of evidence heretofore accumulated, by taking account of differences in LCAT assay procedure and characteristics of the study subjects (general population, high-risk cases of cardiovascular disease, gender) and of the expression profile of the LCAT gene in the pathophysiological state [29, 30]. In fact, no unified view has been obtained so far, based on carotid artery ultrasound study reports, as to the relation of the serum LCAT activity with progression/suppression of atherosclerosis [2, 31, 32].

In this study, we investigated the factors involved in the progression of atherosclerosis with our attention focused on the serum LCAT activity, TPL-related makers and LDL particle size. However, atherosclerosis progresses through a complex network also involving other elements than the above-mentioned factors alone [33]. Further verification of the results of this study will have to take into consideration these other factors as well.

## Conclusions

In this longitudinal study, we confirmed that increased LCAT activity, measured in terms of the serum cholesterol esterification rate by the endogenous substrate method, might be associated with a decrease of the LDL-particle size through its association with an increase in the serum levels of TRL-related markers, which represents disordered TG metabolism. The causal relationship between increase of the LCAT activity and reduction of the LDL-particle size may be determined more clearly in our study due to its longitudinal study design. Thus, measurement of the LCAT activity may be useful for predicting ASCVD in patients at a high risk for progression of atherosclerosis.

## Abbreviations

ANOVA: Analysis of variance
apo: apolipoprotein
ASCVD: Atherosclerotic cardiovascular disease
BMI: Body mass index
CAD: Coronary artery disease
CKD: Chronic kidney disease
DM: Diabetes mellitus
eGFR: estimated glomerular filtration rate
Hb: Hemoglobin
HDL: High-density lipoprotein
hs-CRP: high-sensitivity C-reactive protein
LCAT: Lecithin-cholesterol Acyltransferase
LDL-C: Low-density lipoprotein cholesterol
LDL-Rm: Relative LDL migration
MDRD: Modification of Diet in Renal Disease
RCT: Reverse cholesterol transport
RLP: remnant-like particle
sd: small-dense
SD: Standard deviation
TC: Total cholesterol
TG: Triglyceride
TRL: TG-rich lipoprotein
VLDL: Very-low density lipoprotein

## References

[1] Glomset JA (1968). The plasma lecithins:cholesterol acyltransferase reaction. J Lipid Res.

[2] Dullaart RP, Perton F, Sluiter WJ, de Vries R, van Tol A (2008). Plasma lecithin: cholesterol acyltransferase activity is elevated in metabolic syndrome and is an independent marker of increased carotid artery intima media thickness. J Clin Endocrinol Metab.

[3] Murakami T, Michelagnoli S, Longhi R, Gianfranceschi G, Pazzucconi F, Calabresi L, Sirtori CR, Franceschini G (1995). Triglycerides are major determinants of cholesterol esterification/transfer and HDL remodeling in human plasma. Arterioscler Thromb Vasc Biol.

[4] Sutherland WH, Temple WA, Nye ER, Herbison PG (1979). Lecithin:cholesterol acyltransferase activity, plasma and lipoprotein lipids and obesity in men and women. Atherosclerosis.

[5] Tani S, Takahashi A, Nagao K, Hirayama A (2016). Association of lecithin-cholesterol acyltransferase activity measured as a serum cholesterol esterification rate and low-density lipoprotein heterogeneity with cardiovascular risk: a cross-sectional study. Heart Vessel.

[6] Miller M, Stone NJ, Ballantyne C, Bittner V, Criqui MH, Ginsberg HN, Goldberg AC, Howard WJ, Jacobson MS, Kris-Etherton PM, Lennie TA, Levi M, Mazzone T, Pennathur S, American Heart Association Clinical Lipidology, Thrombosis, and Prevention Committee of the Council on Nutrition, Physical Activity, and Metabolism; Council on Arteriosclerosis, Thrombosis and Vascular Biology, Council on Cardiovascular Nursing, Council on the Kidney in Cardiovascular Disease (2011). Triglycerides and cardiovascular disease: a scientific statement from the American Heart Association. Circulation.

[7] Imai E, Horio M, Nitta K, Yamagata K, Iseki K, Hara S, Ura N, Kiyohara Y, Hirakata H, Watanabe T, Moriyama T, Ando Y, Inaguma D, Narita I, Iso H, Wakai K, Yasuda Y, Tsukamoto Y, Ito S, Makino H, Hishida A, Matsuo S (2007). Estimation of glomerular filtration rate by the MDRD study equation modified for Japanese patients with chronic kidney disease. Clin Exp Nephrol.

[8] Nagasaki T, Akanuma Y (1997). A new colorimetric method for the determination of plasma lecithin-cholesterol acyltransferase activity. Clin Chim Acta.

[9] Bartholome M, Niedmann D, Wieland H, Seidel D (1981). An optimized method for measuring lecithin : cholesterol acyltransferase activity, independent of the concentration and quality of the physiological substrate. Biochim Biophys Acta.

[10] Kobori K, Saito K, Ito S, Kotani K, Manabe M, Kanno T (2002). A new enzyme-linked immunosorbent assay with two monoclonal antibodies to specific epitopes measure human lecithin-cholesterol acyltransferase. J Lipid Res.

[11] DeLong DM, DeLong ER, Wood PD, Lippel K, Rifkind BM (1986). A comparison of methods for the estimation of plasma low- and very low-density lipoprotein cholesterol. The Lipid Research Clinics Prevalence Study. JAMA.

[12] Nakano T, Inoue I, Seo M, Takahashi S, Awata T, Komoda T, Katayama S (2009). Rapid and simple profiling of lipoproteins by polyacrylamide-gel disc electrophoresis to determine the heterogeneity of low-density lipoproteins (LDLs) including small, dense LDL. Recent Pat Cardiovasc Drug Discov.

[13] Hirano T, Ito Y, Yoshino G (2005). Measurement of small dense low-density lipoprotein particles. J Atheroscler Thromb.

[14] Tani S, Matsumoto M, Nagao K, Hirayama A (2014). Association of triglyceride-rich lipoproteins-related markers and low-density lipoprotein heterogeneity with cardiovascular risk: effectiveness of polyacrylamide-gel electrophoresis as a method of determining low-density lipoprotein particle size. J Cardiol.

[15] Tani S, Takahashi A, Nagao K, Hirayama A (2015). Effect of dipeptidyl peptidase-4 inhibitor, vildagliptin on plasminogen activator inhibitor-1 in patients with diabetes mellitus. Am J Cardiol.

[16] Hiro T, Kimura T, Morimoto T, Miyauchi K, Nakagawa Y, Yamagishi M, Ozaki Y, Kimura K, Saito S, Yamaguchi T, Daida H, Matsuzaki M, JAPAN-ACS Investigators (2009). Effect of intensive statin therapy on regression of coronary atherosclerosis in patients with acute coronary syndrome: a multicenter randomized trial evaluated by volumetric intravascular ultrasound using pitavastatin versus atorvastatin (JAPAN-ACS [Japan assessment of pitavastatin and atorvastatin in acute coronary syndrome] study). J Am Coll Cardiol.

[17] Fielding CJ, Fielding PE (1981). Regulation of human plasma lecithin: cholesterol acyltransferase activity by lipoprotein acceptor cholesteryl ester content. J Biol Chem.

[18] Chung BH, Segrest JP, Franklin F (1998). In vitro production of beta-very low density lipoproteins and small, dense low density lipoproteins in mildly hypertriglyceridemic plasma: role of activities of lecithin:cholester acyltransferase, cholesterylester transfer proteins and lipoprotein lipase. Atherosclerosis.

[19] Hutchins PM, Heinecke JW (2015). Cholesterol efflux capacity, macrophage reverse cholesterol transport and cardioprotective HDL. Curr Opin Lipidol.

[20] Dobiásová M, Frohlich J (1998). Understanding the mechanism of LCAT reaction may helpto explain the high predictive value of LDL/HDL cholesterol ratio. Physiol Res.

[21] Dobiasova M, Stribrna J, Sparks DL, Pritchard PH, Frohlich JJ (1991). Cholesterol esterification rates in very low density lipoprotein- and low density lipoprotein-depleted plasma. Relation to high density lipoprotein subspecies, sex, hyperlipidemia, and coronary artery disease. Arterioscler Thromb.

[22] Tani S, Matsuo R, Kawauchi K, Yagi T, Atsumi W, Hirayama A (2018). A cross-sectional and longitudinal study between association of n-3 polyunsaturated fatty acids derived from fish consumption and high-density lipoprotein heterogeneity. Heart Vessel.

[23] Gebhard C, Rhainds D, He G, Rodés-Cabau J, Lavi S, Spence JD, Title L, Kouz S, L’Allier PL, Grégoire J, Ibrahim R, Cossette M, Guertin MC, Beanlands R, Rhéaume E, Tardif JC (2018). Elevated level of lecithin:cholesterol acyltransferase (LCAT) is associated with reduced coronary atheroma burden. Atherosclerosis.

[24] Tanaka S, Yasuda T, Ishida T, Fujioka Y, Tsujino T, Miki T, Hirata K (2013). Increased serum cholesterol esterification rates predict coronary heart disease and sudden death in a general population. Arterioscler Thromb Vasc Biol.

[25] Andrews JPM, Fayad ZA, Dweck MR (2018). New methods to image unstable atherosclerotic plaques. Atherosclerosis.

[26] Daniels JA, Mulligan C, McCance D, Woodside JV, Patterson C, Young IS, McEneny J (2014). A randomised controlled trial of increasing fruit and vegetable intake and how this influences the carotenoid concentration and activities of PON-1 and LCAT in HDL from subjects with type 2 diabetes. Cardiovasc Diabetol.

[27] Iida K, Tani S, Atsumi W, Yagi T, Kawauchi K, Matsumoto N, Hirayama A (2017). Association of plasminogen activator inhibitor-1 and low-density lipoprotein heterogeneity as a risk factor of atherosclerotic cardiovascular disease with triglyceride metabolic disorder: a pilot cross-sectional study. Coron Artery Dis.

[28] Asztalos BF, Swarbrick MM, Schaefer EJ, Dallal GE, Horvath KV, Ai M, Stanhope KL, Austrheim-Smith I, Wolfe BM, Ali M, Havel PJ (2010). Effects of weight loss, induced by gastric bypass surgery, on HDL remodeling in obese women. J Lipid Res.

[29] Ossoli A, Simonelli S, Vitali C, Franceschini G, Calabresi L (2016). Role of LCAT in atherosclerosis. J Atheroscler Thromb.

[30] Calabresi L, Simonelli S, Gomaraschi M, Franceschini G (2012). Genetic lecithin:cholesterol acyltransferase deficiency and cardiovascular disease. Atherosclerosis.

[31] Calabresi L, Baldassarre D, Simonelli S, Gomaraschi M, Amato M, Castelnuovo S, Frigerio B, Ravani A, Sansaro D, Kauhanen J, Rauramaa R, de Faire U, Hamsten A, Smit AJ, Mannarino E, Humphries SE, Giral P, Veglia F, Sirtori CR, Franceschini G, Tremoli E (2011). Plasma lecithin:cholesterol acyltransferase and carotid intima-media thickness in European individuals at high cardiovascular risk. J Lipid Res.

[32] Hovingh GK, Hutten BA, Holleboom AG, Petersen W, Rol P, Stalenhoef A, Zwinderman AH, de Groot E, Kastelein JJ, Kuivenhoven JA (2005). Compromised LCAT function is associated with increased atherosclerosis. Circulation.

[33] Ross R (1999). Atherosclerosis—an inflammatory disease. N Engl J Med.

